# Provenance and distribution of zinc in terrestrial planets

**DOI:** 10.1038/s41598-025-24419-4

**Published:** 2025-11-18

**Authors:** Rayssa Martins, Elin M. Morton, Yihang Huang, Helen M. Williams, Mark Rehkämper

**Affiliations:** 1 Department of Earth Sciences and Engineering, Imperial College London, London, UK; 2 Department of Earth Sciences, The University of Cambridge, Cambridge, UK

**Keywords:** Early solar system, Inner planets

## Abstract

The nucleosynthetic Zn isotope compositions of planetary materials shed light on the sources of Zn and other similarly volatile elements during terrestrial planet formation. Previous results for Earth revealed that it acquired its Zn inventory primarily from primitive, undifferentiated bodies originating from both the inner and outer Solar System. To further investigate this, we obtained new nucleosynthetic Zn isotope data for six Martian meteorites and five Lunar samples. Our Martian data are consistent with previous findings which show that, despite being more volatile-rich than Earth, Mars’s Zn was derived almost entirely from inner Solar System sources. We incorporated these results into a comprehensive mixing model, including eight additional isotope systems and major element abundances. The model suggests that Mars accreted ~ 50% of its mass from primitive chondritic material that delivered ~ 90% of its Zn, while the remainder came from volatile-poor, differentiated bodies. This mirrors previous findings for Earth, where ~ 90% of Zn was delivered by primitive material, despite contributing only ~ 30% of Earth’s total mass. Together, these results imply that the volatile inventories of terrestrial planets depend more on the nature (primitive versus differentiated) than the origin (inner versus outer Solar System) of their building blocks. Finally, the nucleosynthetic Zn isotope compositions of five Lunar samples are indistinguishable, within uncertainty, from those of the bulk silicate Earth and non-carbonaceous meteorites. Further analyses are thus needed to more robustly constrain the origin of Lunar Zn.

**Supplementary Information:**

The online version contains supplementary material available at 10.1038/s41598-025-24419-4.

## Introduction

Constraining the origins of volatiles in terrestrial planets is essential for understanding their formation and how they become habitable. Recent nucleosynthetic isotope studies showed that Earth’s inventories of the moderately volatile elements Zn and K are derived from mixed sources, including planetesimals that formed locally in the inner Solar System as well as material that originated from much greater heliocentric distances^1–4^. Furthermore, accretion of primitive chondritic material, which was not impacted by melting and differentiation in the early Solar System, has been shown to play a crucial role in establishing Earth’s volatile content^5^. Nonetheless, it is unclear whether these features are common across terrestrial planets, or whether Earth presents a special case.

Early studies of Mars attempted to reproduce its mantle composition, as inferred from SNC meteorites, by mixing of nebular components with different volatile abundances^6,7^. In contrast, later work primarily applied O isotopes and nucleosynthetic isotope data for refractory elements to constrain the meteorite endmembers which contributed to Mars’ accretion, and typically inferred that Mars formed to ≥ 90% by mass from non-carbonaceous (NC) materials sourced from the inner Solar System. However, when using only chondritic endmembers, such models produced bulk elemental compositions that were too volatile-rich^8–10^. In accord with these findings, recent nucleosynthetic Zn isotope investigations showed that Mars’ Zn budget was supplied primarily by locally sourced NC material^11,12^, but the contribution of primitive chondritic material to Martian volatiles remained unconstrained. Importantly, the extent of volatile depletion exhibited by a planetesimal or planet may not exclusively reflect nebular processes, as substantial volatile loss can occur during accretion. Heat derived from the decay of extinct radionuclides can produce enough energy to induce the formation of magma oceans in small, differentiated planetesimals, which allows for effective volatilization and degassing^13^. These processes furthermore produce mass-dependent isotope fractionations consistent with those observed in some planetesimals^14,15^. Therefore, when planets accrete from a mixture of differentiated and undifferentiated materials, the mass balance of the mixture will shape their volatile inventories.

Albeit not directly, the composition and volatile content of a third terrestrial body can be investigated – Theia, the Mars-sized planet that likely collided with proto-Earth to form the Moon^16^. Theia’s existence is inferred from the giant impact hypothesis, the most prominent theory for the formation of the Moon^17,18^. While the Moon is generally thought to have formed primarily from the impactor, Theia’s composition remains elusive, as it cannot be directly accessed. Constraints are available from the similarities in the nucleosynthetic isotope compositions of the Earth and Moon^19–22^ but nucleosynthetic isotope data for volatile elements in Lunar samples are currently lacking, due to the challenges posed by analyses of small, volatile-depleted samples of limited availability. Here, we present mass-independent Zn isotope results for Martian and Lunar samples, in an attempt to better constrain the volatile sources of the terrestrial planets.

## Results

**Fig. 1:**
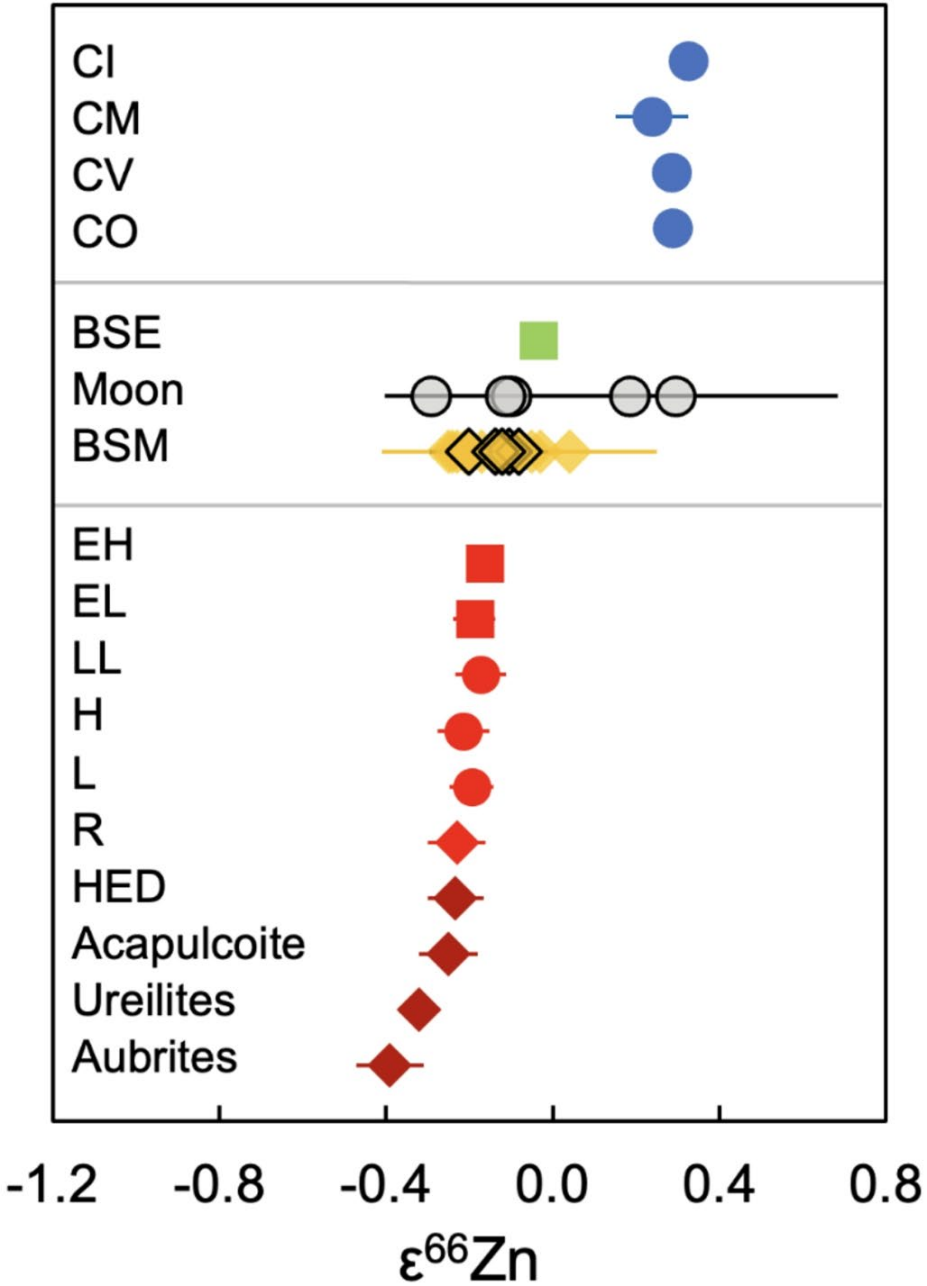
Mass-independent Zn isotope compositions for CC and NCC meteorites in comparison to the BSE, Moon and Mars (BSM). The data for Mars overlap with NCC values for ordinary (OC), enstatite (EC), and Rumuruti (R) chondrites, but are clearly distinct from CCs. The lunar samples display variable results. All data are compiled in the Supplementary Data File, with the results of this study presented by outlined symbols.

The study investigated six Martian meteorites, three Apollo Lunar samples, and two Lunar meteorites (Table 1). For quality control, an aliquot of the Orgueil CI1 meteorite and the USGS geological reference material BCR-2 were analyzed concurrently. For the latter, the measured εZn values are indistinguishable from the London Zn isotope standard, whilst the data obtained for Orgueil are consistent with previously published values for this meteorite (Table 1). The Martian meteorites all display resolvable anomalies relative to the terrestrial composition, yielding results for bulk silicate Mars (BSM), with ε^66^Zn_BSM_ = −0.15 ± 0.05 (2se; Fig. 1; Table 1) in agreement with previous data^11,12^. Additional mass-dependent Zn isotope analyses for five of the six Martian meteorites with the double spike technique (see Supplementary Information) yielded δ^66^Zn values between 0.32 ± 0.03 and 0.54 ± 0.02, to define a mean δ^66^Zn of 0.42 ± 0.15 for BSM, also in accord with previous findings^12^ (Table 1).

**Table 1:** Mass-independent Zn isotope data (in εZn notation) for Martian and lunar samples, as well as orgueil CI1 and BCR-2; the data were obtained from ^i^Zn/^68^Zn ratios employing ^64^Zn/^68^Zn for internal normalization. Also shown are δ^66^Zn values determined for the Martian samples.

Sample	Type	Mass (g)	ε^66^Zn	2sd	2se	ε^67^Zn	2sd	2se	*n*	δ^66^Zn	2sd	*n*
MIL 03346	Nakhlite	0.31	-0.26	0.32	0.12	0.05	0.32	0.12	7	0.32	0.03	4
ALH 77005	Shergottite	0.29	-0.10	0.26	0.09	0.04	0.42	0.15	8	0.54	0.02	4
EET 79001	Shergottite	0.3	-0.20	0.23	0.09	-0.03	0.39	0.16	6	0.40	0.06	4
LAR 12095	Shergottite	0.3	-0.08	0.21	0.07	0.16	0.21	0.08	7	0.45	0.02	4
RBT 04262	Shergottite	0.3	-0.14	0.18	0.07	0.06	0.22	0.09	6	0.38	0.04	5
ALH 84001	SNC OPX	0.3	-0.12	0.23	0.07	0.10	0.32	0.09	12	-	-	-
*BSM*			-0.15	0.12	0.05	0.06	0.12	0.05	6	0.42	0.15	5
70017-587	High-Ti mare basalt	1.898	-0.11	0.21	0.07	0.03	0.22	0.07	9	-	-	-
10017-423	High-Ti ilmenite basalt	0.4	0.29	0.87	0.39	0.44	0.48	0.21	5	-	-	-
15016-254	Low-Ti olivine basalt	2.4	-0.10	-	0.30	0.02	-	0.17	2	-	-	-
NWA 11182	Feldspathic breccia	2.0	0.18	-	0.19	0.40	-	0.11	2	-	-	-
NWA 11898	Feldspathic breccia	2.05	-0.29	0.13	0.06	0.17	0.08	0.04	4	-	-	-
*Lunar mean*			-0.01	0.43	0.19	0.14	0.48	0.21	5	-	-	-
Orgueil	CI1		0.32	0.24	0.06	-0.07	0.25	0.06	18	-	-	-
BCR-2	Terrestrial		0.00	0.26	0.04	0.00	0.27	0.05	34	-	-	-

n is the total number of individual analytical runs for a given sample, for one or several powder/digest solution aliquots. The reported 2sd and 2se uncertainties for samples with *n* > 2 are based on the results obtained for n individual runs; for samples with *n* = 2, only a 2se error is reported, which reflects the average within-run uncertainty of the two sample runs.

**Fig. 2:**
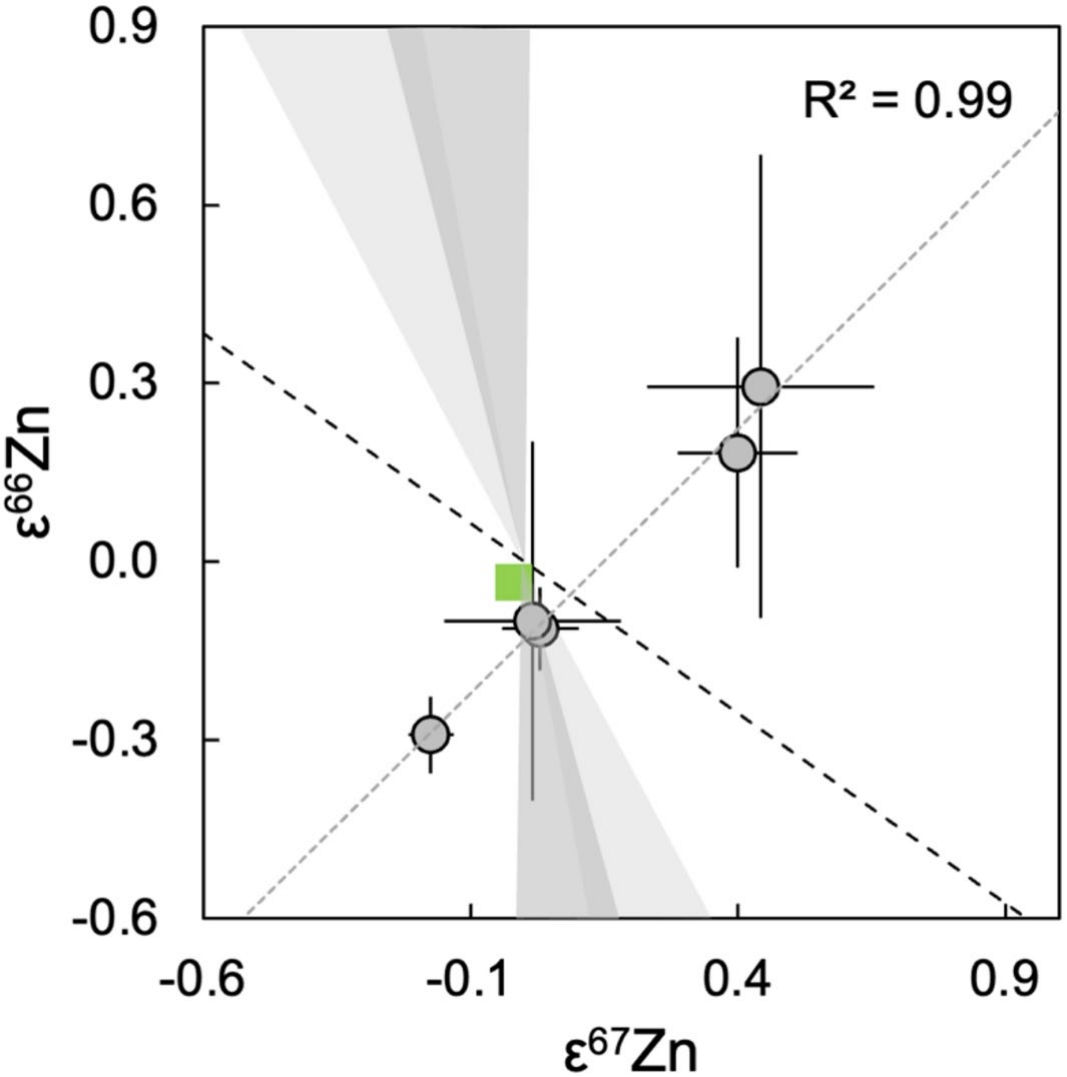
Plot of ε^66^Zn versus ε^67^Zn values for the five Lunar samples analyzed in this study (grey), with the BSE composition (green) shown for comparison. The Lunar εZn values define a correlation which is distinct from the trends predicted for Zn production in ECSNe (light grey field) and SNIa (dark grey field) supernovae, as modelled following Martins et al.^1^. The isotopic shifts from possible nuclear field shift effects (dashed black line) are also shown^1^.

Due to the limited sample masses and their severe Zn depletion, the mass-independent Zn isotope analyses of the Lunar samples used run solutions with Zn concentrations as low as 150 ng g^− 1^ (compared to up to 500 ng g^− 1^ for other samples), employed fewer data acquisition cycles, and encompassed fewer repeat runs than were possible for the other samples. This resulted in εZn values with larger uncertainties compared to the other data presented in this study (Table 1). The results for 15016 and 70017 are indistinguishable from terrestrial Zn and NC meteorites, with ε^66^Zn = −0.10 ± 0.30 and −0.11 ± 0.07, respectively (Table 1; Fig. 2). In contrast, NWA 11898 displays resolvable negative anomalies relative to the terrestrial mean within ± 2se (ε^66^Zn = −0.29 ± 0.06), while NWA 11182 and 10017 display resolvable positive anomalies, with ε^67^Zn values of 0.40 ± 0.11 and 0.44 ± 0.21, respectively. Finally, the mean obtained for the five Lunar samples, with ε^66^Zn_Lunar_ = 0.01 ± 0.19 (2se) overlaps with previously attained values for the BSE and NC chondrites^1^ (NCCs; Table 1; Fig. 1).

## Discussion

### Lunar samples

Whilst lunar samples display identical mass-independent isotope compositions to the BSE for several isotope systems, including O, Ca, Ti, Cr, Zr and W^19–25^, most recent dynamical models of the giant impact suggest that proto-Earth’s contribution to the Moon was limited, with approximately 70–80% of the Lunar mass derived from the giant impactor^16,26^. Combined, these observations may imply that the isotopic similarities also extended to Theia. However, the likelihood of an accretion scenario where Theia was isotopically identical to the BSE for most isotope systems is considered to be minimal^26^. This constraint is even more stringent for Zn, given the large differences in Zn concentrations amongst and between CCs, NCCs, and differentiated NC parent bodies (DNCs)^27–31^. As a result, even if Theia and Earth accreted from the same proportions of these materials, differences in their Zn abundances could still produce distinct isotope compositions.

The improbability of a scenario where Theia was isotopically identical to the BSE led to the hypothesis of post-impact homogenization^32,33^. The latter scenario allows for distinct compositions for the impactor and the BSE, but which would have been homogenized through vapor-phase equilibration between Earth’s mantle and the material from which the Moon formed. Previous studies have shown that such a scenario is feasible even for refractory elements, such as Ti^22^. Such a process should therefore inevitably affect Zn, as this is volatile and, hence, more susceptible to vaporization and re-equilibration.

The five Lunar samples display variable compositions, ranging from ε^66^Zn = −0.29 ± 0.06 to ε^66^Zn = 0.29 ± 0.39 (2se; Fig. 1; Table 1). All samples furthermore present a well-defined correlation between ε^66^Zn and ε^67^Zn values (R^2^ = 0.99; Fig. 2). Importantly, the slope of this correlation is incompatible with variations predicted for nucleosynthetic Zn isotope anomalies originating from either electron capture core collapse (ECSNe) or Type Ia (SN Ia) supernovae, which have been shown to be consistent with the anomalies of other Solar System materials^1^ (Fig. 2). It is hence likely that the observed variations reflect other processes that can affect mass-independent Zn isotope compositions, and this is assessed in the following.

It is conceivable that the εZn data obtained for some samples are impacted by isobaric or molecular interferences, or matrix effects from unwanted constituents in the analyzed sample solutions. To test this, elemental analyses of the run solutions used for the Zn isotope measurements were performed with a quadrupole ICP-MS instrument. Small aliquots of the three Apollo samples, Orgueil, and BCR-2 were taken after the Zn purification procedure. No systematic and significant differences were detected between any of the samples, which displayed concentrations comparable to the blank for all 57 elements that were analyzed. An aliquot of sample 10017 was furthermore subjected to a digestion protocol that employed hydrogen peroxide in an attempt to eliminate organic constituents possibly leached from the ion exchange resins utilized for the purification of Zn^34^. This resulted in no detectable differences in the mass-independent isotope composition of this sample (ε^66^Zn = 0.58 ± 0.18; ε^67^Zn = 0.53 ± 0.10) from the mean obtained for aliquots that were not subjected to this procedure (ε^66^Zn = 0.22 ± 0.46; ε^67^Zn = 0.42 ± 0.26).

An alternative origin for the unexpected mass-independent Lunar Zn isotope data is that the samples were impacted by mass-dependent isotope fractionations, either in nature or the laboratory, which were not adequately corrected by the exponential law, which was employed for internal normalization of the εZn data. To evaluate this in the absence of dedicated mass-dependent isotope analyses (due to the limited Zn available), the δ^66^Zn values for the samples were determined from the data that had been collected for the measurement of εZn. This involved determination of δ^66^Zn from the ^66^Zn/^64^Zn ratios measured for samples without internal normalization of the mass bias, relative to results obtained for bracketing runs of the Zn isotope standard (see Supplementary Information for details). Given this procedure, the Lunar δ^66^Zn values, summarized in Supplementary Table S1, are less precise and less robust than the Martian data, which were obtained with established methods on dedicated sample aliquots.

The δ^66^Zn values of the five lunar samples are highly variable, with results between − 6.0 ± 0.2 for Apollo basalt 10017 and 9.2 ± 0.2 for the felspathic lunar meteorite NWA 11898 (Supplementary Table S1). In a diagram of δ^66^Zn versus ε^66^Zn (Supplementary Fig. S1A), the samples, furthermore, appear to display a correlation that could indicate a causal relationship between the isotope data. However, a number of observations argue against this interpretation. It is conceivable that the correlation might be caused by laboratory-induced isotope fractionation, particularly during Zn purification by ion exchange chromatography. This is unlikely, however, because all non-lunar samples analyzed thus far in our laboratory yielded highly reproducible εZn data that are not impacted by the effects recorded by the Lunar samples^1,5,35^. In addition, the δ^66^Zn values determined here for the three Apollo rocks deviate from previously published results for different aliquots of the same samples by less than 0.9‰ (Supplementary Table S1). This demonstrates that processing of the Lunar samples in our laboratory was not associated with mass fractionation effects large enough to significantly impact the measured εZn values.

The conclusion that the δ^66^Zn variations of our Lunar samples are (primarily) of natural origin is supported by previous studies, which identified similar mass-dependent Zn isotope variations in lunar rocks^36,37^. The observed heavy and light Lunar Zn isotope compositions were thereby generally ascribed to partial evaporation and partial condensation of the element, respectively, at Rayleigh conditions^36–38^. Modeling of the expected isotope fractionation shows that the high ε^66^Zn observed for 10017 could be an artefact of partial kinetic Rayleigh condensation of Zn, due to the low measured δ^66^Zn (Supplementary Table S1), but additional evidence for this speculative interpretation is lacking. Furthermore, other data argue against a role of such artefacts. First, both kinetic and equilibrium evaporation of Zn under Rayleigh conditions would generate high δ^66^Zn values coupled with higher ε^66^Zn. This prediction, however, stands in contrast to the high δ^66^Zn and low ε^66^Zn determined for lunar meteorite NWA 11898 (Supplementary Table S1). As such, the low ε^66^Zn of this sample is not an artefact of the fractionated mass-dependent isotope composition and, overall, the correlated δ^66^Zn-ε^66^Zn variations seen in Supplementary Fig. S1A are most likely a spurious result. Second, the Apollo samples 70017 and 15016 differ in their δ^66^Zn values by almost 2.5‰, but they are identical in ε^66^Zn and ε^67^Zn, within error (Supplementary Table S1). Considering the latter observations, it appears unlikely that the mass-independent Zn isotope compositions of the Lunar rocks are significantly impacted by mass-dependent isotope fractionations. In addition, the results are also incompatible with modeled compositions for mass-independent isotope fractionation caused by the nuclear field shift effects (Fig. 2)^1^.

All samples are derived from the Lunar surface, where they are exposed to galactic cosmic rays, which could, in principle, alter Zn isotope compositions. Whilst still comparatively small, ^67^Zn has the largest neutron capture cross-section among the five Zn isotopes, and cosmogenic reactions could hence decrease the abundance of ^67^Zn through an induced (n,γ) reaction to produce ^68^Zn. The potential effects of this process can be most readily assessed by examining the ε^67^Zn values determined for the samples. Although sample 10017 displays the largest offset from the mean (ε^67^Zn = 0.44 ± 0.21, 2se) and the highest cosmogenic exposure age (480 ± 70 Ma)^39^, the positive ε^67^Zn value is the opposite of what would be expected for a decrease in the abundance of ^67^Zn caused by cosmogenic effects. Furthermore, the two other Apollo samples with identical ε^67^Zn values (ε^67^Zn = 0.03 ± 0.07; ε^67^Zn = 0.02 ± 0.16) have clearly distinct cosmogenic exposure ages, of 315 ± 20 and 220 ± 20 Ma, for 15016 and 70017, respectively^40,41^ (Supplementary Fig. S1B). Therefore, even if present, the effects of cosmogenic exposure on the mass-independent Zn isotope compositions of the analyzed samples are unlikely to be resolvable within the obtained precision.

Samples from the Lunar surface may also be contaminated by local impacting material that originates from isotopically distinct bodies. Previous studies found no substantial evidence for variations in the nucleosynthetic isotope composition of Lunar samples for other isotope systems^19,23^. However, Zn may be more susceptible to such alteration due to the severe Zn depletion recorded by the Moon. Nonetheless, as stated previously, the compositions of the Lunar samples define an excellent correlation, with a slope that is clearly distinct from the nucleosynthetic isotope variations predicted for the potential sources of the nucleosynthetic Zn anomalies (Fig. 2). The variability in the mass-independent Zn isotope composition of the Lunar samples is therefore unlikely to reflect contamination from isotopically distinct materials.

Hence, while it is unclear which processes are recorded by the mass-independent Zn isotope composition of the Lunar samples analyzed in this study, it is unlikely that they all reflect the nucleosynthetic isotope composition of the Moon. As the only samples consistent with predicted nucleosynthetic isotope compositions, 15016 and 70017 are the least likely to have been affected by other processes and may in fact reflect the nucleosynthetic Zn isotope composition of the Moon. If so, this would define a mean nucleosynthetic Zn isotope composition for the Moon that is indistinguishable from the BSE (ε^66^Zn = −0.10 ± 0.30; ε^67^Zn = 0.02 ± 0.17), in line with findings for other isotope systems. However, with the available precision, the results are also indistinguishable from NCs (Fig. 1). Further analyses are hence required to confirm whether no distinctions can be resolved between the Lunar samples and the BSE.

### Martian samples

As for non-volatile elements, the Martian samples analyzed in this study display resolvable nucleosynthetic Zn isotope anomalies relative to the BSE, with ε^66^Zn_BSM_ = −0.15 ± 0.05 (2se), in line with previous studies^11,12^. The results for Mars furthermore overlap with data for NCCs^1,3,4^ (Fig. 2). To estimate the CC-derived Zn contribution to Mars’ Zn inventory, mixtures between different CC groups and NC groups, encompassing both NCCs and DNCs, were modelled using a Monte Carlo simulation with 10,000 trials (detailed information on the modelling is available in the Supplementary Information). In the first run, all 17 meteorites groups for which nucleosynthetic Zn isotope compositions are available were included (see Supplementary Data File). For each trial, the endmembers were randomly selected, and their compositions were generated in a normal distribution within the adopted values, along with arbitrary mass fractions between 0 and 100%. The final compositions of the mixtures were calculated by mass balance but only results which yielded Mars-like ε^66^Zn values were selected (ε^66^Zn_BSM_ = −0.15 ± 0.03, 2se; Table 1 and Supplementary Data File).

**Fig. 3:**
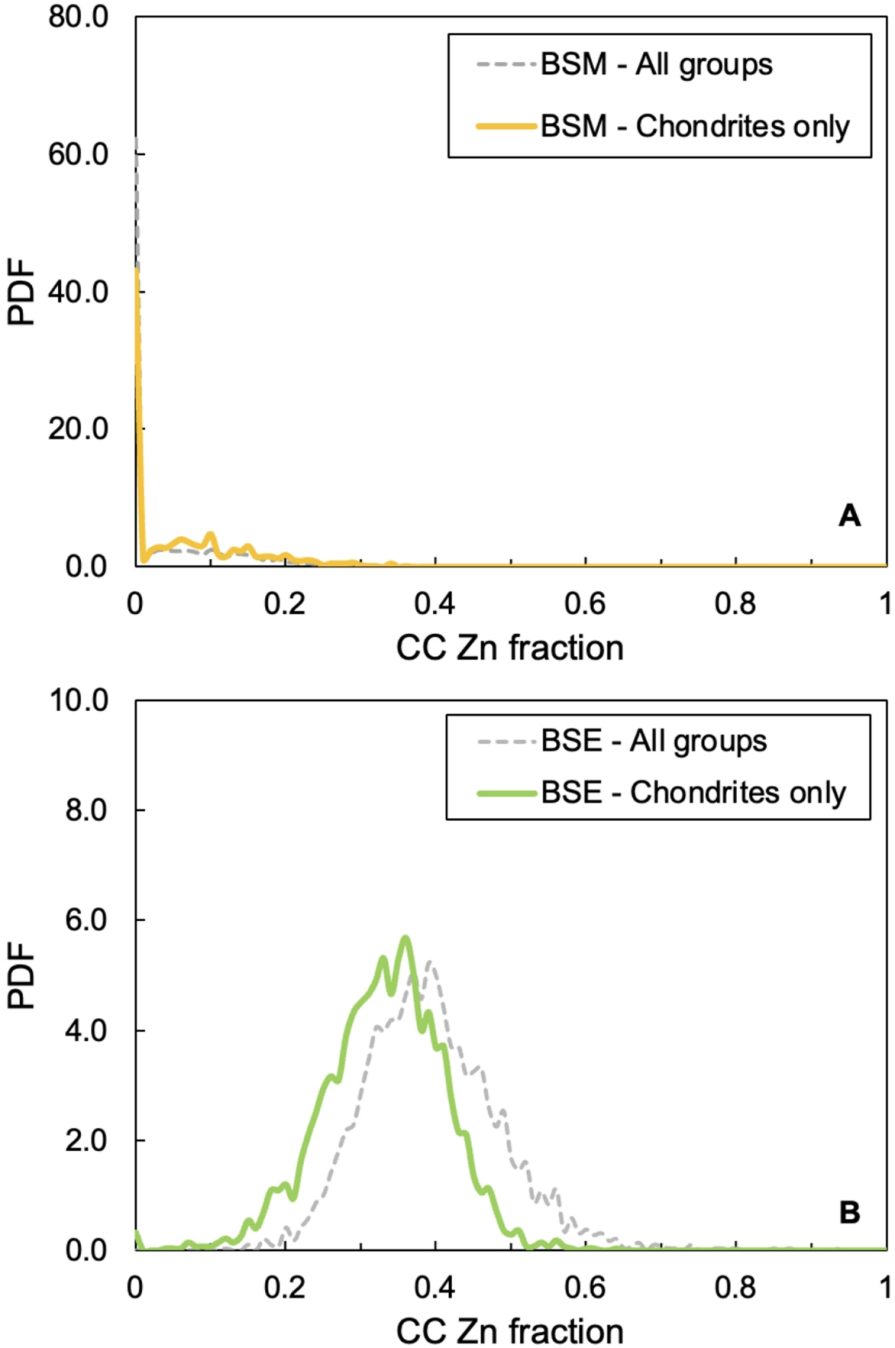
Modelled Zn mass fractions derived from CC material for Mars and Earth. (**A**) Probability density functions resulting from two Monte Carlo simulations that reproduce the nucleosynthetic Zn isotope compositions of BSM using all meteorite groups (dashed grey line) and only chondrites (yellow line). Most successful trials correspond to a CC Zn fraction of zero. (**B**) Same models as in (**A**), but for the BSE. The most common combination that resulted in BSE-like compositions required ~ 40% and ~ 36% CC-like Zn, if all groups (dashed grey line) or only chondrites (green line) were employed, respectively.

Most successful trials (63%) correspond to mixtures where no CC-derived Zn was included, indicating that Mars’ nucleosynthetic Zn isotope composition can be more readily reproduced by exclusively incorporating Zn from NC material (Fig. 3). The average contribution of CC-like Zn to Mars for all successful trials is ~ 16%, with 35% of trials falling in the range ~ 1–20%. These results agree with previous models, which estimated a CC Zn contribution to Mars of ~ ﻿23%^12^ and up to ~ 22%^11^. However, differentiated NC material in the form of eucrite and angrite achondrites was included in this modeling, even though such material was estimated to have supplied just ~ 10% of Earth’s Zn^5^. As the Zn contributions from NC achondrites to Mars were also likely very limited, another run of the model was performed where only chondritic groups were included as endmembers. In this case, 43% of successful trials included no CC-derived Zn, and 51% included between 1 and 20% CC-like Zn. As such, the inclusion of differentiated meteorite groups had only minor effects on the outcome of the model.

For comparison, the same models were applied to the BSE (ε^66^Zn = −0.03 ± 0.03, 2se; Supplementary Data File). When all groups were included, trials with ~ 20–65% CC-like Zn were successful, with most results falling at ~ 40%, in line with previous studies^1,3,5,11^. In the run that employed only chondrites, most of the successful trials fall between ~ 15–55%, with a maximum probability at ~ 36% CC-derived Zn. (Fig. 3). In either case, as Mars appears to have received only a negligible Zn contribution from material with a CC origin, our data thus define a much more substantial CC contribution to the BSE than to BSM. The different chondritic sources of Earth’s and Mars’ Zn are hence possibly responsible for the observation that for BSM, the mass dependent Zn isotope data of this study and a previous investigation define a δ^66^Zn value for BSM, of about 0.4 to 0.5 (Table 1)^12^, which is significantly higher compared to current BSE estimates (δ^66^Zn = + 0.16 ± 0.06 to + 0.20 ± 0.05)^42–45^.

In addition to constraining the Zn contribution from CC materials, we also employed our results in a more comprehensive model to identify the main building blocks accreted by Mars. To do so, elemental concentrations were modelled for ten major elements (Na, Mg, Si, P, Ca, K, Ti, Fe, and Mn) and isotope compositions for nine isotope systems (Δ^17^O, ɛ^48^Ca, ɛ^50^Ti, ɛ^54^Cr, ɛ^64^Zn, ɛ^84^Sr, ɛ^96^Zr, δ^30^Si and δ^25^Mg) using the same approach applied by Martins et al.^5^, with further details provided in the Supplementary Information. The applied isotope systems are restricted to lithophile elements, as for siderophile elements the mantle composition is primarily determined by late-accreted material, which could lead to biased results for heterogenous accretion scenarios. The models employed Monte Carlo simulations with variable numbers of trials depending on the run (Table 2). To allow for a direct comparison with the results reported for the BSE by Martins et al.^5^, the current study employs the same modeling approach and parameters, including the use of ɛ^64^Zn values internally normalized to the ^66^Zn/^67^Zn ratio (Table S2). All relevant literature data for Mars are summarized in Tables S3 and S4 (Supplementary information). The endmembers included CC (CI, CM, CV, CO) and NCC (H, L, LL ordinary chondrites and EH, EL enstatite chondrite) meteorite groups, as well as the parent bodies of two non-carbonaceous achondritic meteorites, eucrites and angrites (EPB and APB, respectively), which are derived from DNC planetesimals, with endmember compositions also summarized in Supplementary Tables S3 and S4.

**Table 2:** Summary of results from the Monte Carlo simulations for the Building blocks of Mars.

Run	1	2	3	4
Nucleosynthetic isotope composition	✓		✓	✓
Major elements ratios		✓	✓	✓
Endmembers	CC, NCC, APB, EPB	CC, NCC, APB, EPB	CC, NCC, APB, EPB	CC, NCC, UM1, UM2
Trials	10^8^	10^4^	10^8^	10^8^
Solutions	478	2428	0	787
CC (%, ± 2sd)	4 ± 3	$\:{\text{15}}_{\text{-15}}^{\text{+33}}$	-	1 ± 1
NCC (%, ± 2sd)	87 ± 17	44 ± 29	-	47 ± 23
APB/EPB/UM (%, ± 2sd)	13 ± 11	44 ± 27	-	53 ± 23

Checkmarks indicate literature values for Mars that are successfully reproduced by each run. Meteorite groups included in the models: CI, CM, CO, CV, EH, EL, H, L, LL, eucrite parent body (EPB), angrite parent body (APB), and Unsampled Materials 1 and 2, UM1 and UM2. UM1 and UM2 have eucrite- and angrite-like elemental abundances and mass-dependent isotope compositions, but OC and EC-like nucleosynthetic isotope compositions, respectively, following Martins et al.^5^.

**Fig. 4:**
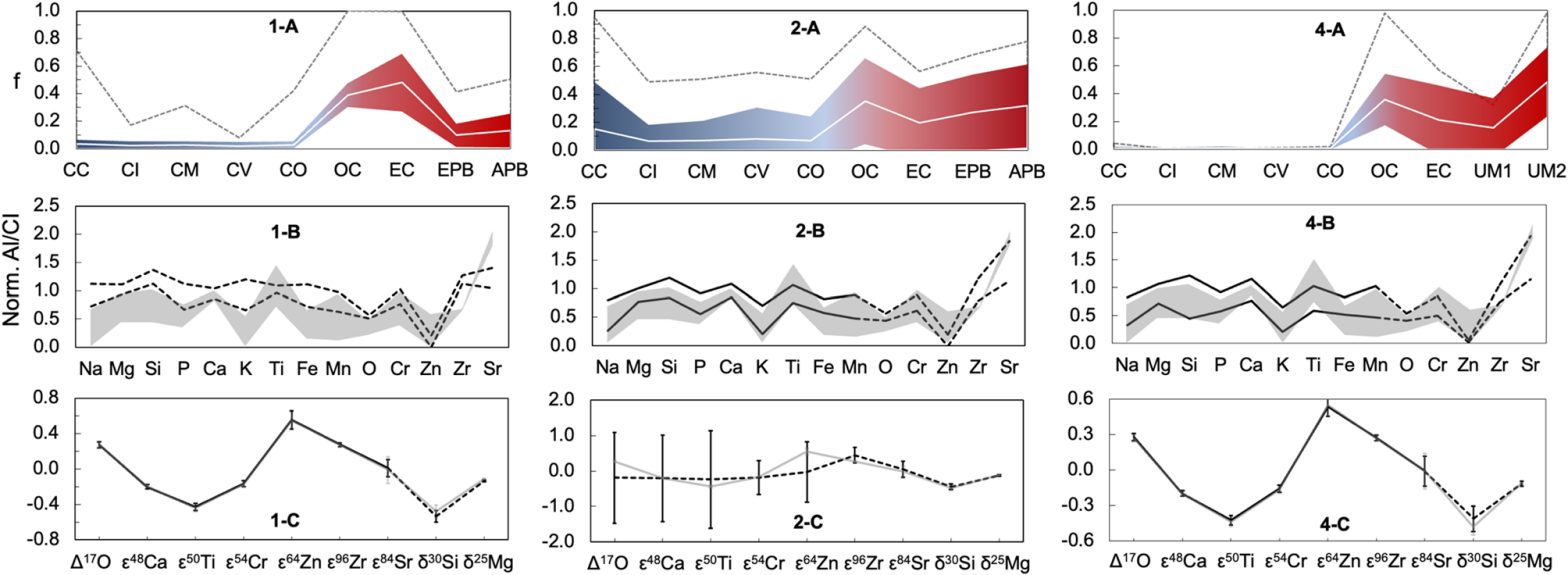
Mixing models that attempt to reproduce the isotope and elemental composition of bulk Mars. The numbers on the panels correspond to the Run number of the simulation (Table 2). (**A**) Mean mass fraction obtained for each meteorite group for all valid solutions (white lines) and respective uncertainties (2sd, colored fields). The dashed lines illustrate the frequency at which each group appears in valid solutions, where f = 1 means that a given group appears in 100% of valid solutions. The CC fraction denotes the sum of all CC groups. (**B**) Ratio of a given element to Al, normalized to CI chondrites. The lines represent the highest and lowest values obtained for all valid solutions, while the grey fields show estimates for Mars (Supplementary Table S4). Elements shown with a solid line were used as criteria for the selection of valid results, while elements with dashed lines were not considered in the evaluation of results. (C) Mean isotope compositions for all valid solutions of each run, with error bars indicating twice the standard deviation. Ratios shown with a solid line were used as criteria for selection of valid results, while ratios with a dashed line were not considered in the evaluation of results. The grey lines denote literature data for Mars (Supplementary Table S3).

In the modelling, the endmembers were randomly selected and assigned arbitrary mass fractions for each of the trials. Their isotope and elemental compositions were randomly generated within the compiled literature values. The resulting compositions of the mixtures were calculated by mass balance and the selection of valid results varied between runs. The mass fractions of each endmember and the resulting isotope compositions are reported as mean values and twice the standard deviation, based on all valid trials for each run (Fig. 4). For the elemental abundances, the highest and lowest values obtained in valid runs are presented in Fig. 4. A total of four runs were performed, with each featuring distinct criteria for the selection of valid solutions (Table 2).

For the first run of the model, only trials that yielded Mars-like mass-independent isotope compositions were selected (Δ^17^O, ɛ^48^Ca, ɛ^50^Ti, ɛ^54^Cr, ɛ^64^Zn, ɛ^84^Sr, and ɛ^96^Zr), and this resulted in a mixture comprised mainly of NCCs (87 ± 17%) with much more limited fractions of CC (4 ± 3%), and DNC material (13 ± 11%). However, this produced elemental abundances that are inconsistent with literature estimates for Mars, with noticeable excesses of volatile elements (e.g., Na, P, K). The elemental abundances were successfully reproduced by Run 2, which yielded a smaller NCC fraction (44 ± 29%) combined with larger contributions from CCs ($\:{15}_{-15}^{+33}$%) and DNCs (44 ± 27%). The solutions resulting from this run, however, produced highly variable mass-independent isotope compositions that were not compatible with literature values; this reflects that no single Run 2 trial that reproduced Mars’ chemical composition was in accord with the constraints imposed by the nucleosynthetic isotope data. Nonetheless, the mass-dependent δ^30^Si and δ^25^Mg values of these mixtures were consistent with literature results for Mars. Finally, Run 3 showed that no combination of these CC, NCC and DNC materials could reproduce both the Martian isotope compositions and the elemental abundances. In accord with the results of other recent modeling studies, this may imply the involvement of material unsampled by meteorites in the accretion of Mars^46,47^.

Combined, the results of Runs 1, 2, and 3 suggest that, in addition to CCs and NCCs, Mars likely accreted a significant fraction of material with NCC-like nucleosynthetic isotope compositions, but which was volatile depleted, akin to the APB and EPB parent bodies. One such body is possibly the parent asteroid of aubrites, a group of meteorites thought to originate from a differentiated enstatite chondrite-like planetesimal^37^. In particular, aubrites have nucleosynthetic O, Ca, Ti, Cr, and Ni isotope compositions which are indistinguishable from ECs but they are substantially more volatile-depleted, with Zn concentrations of 0.2 to 13 µg^− 1^^48^. However, s-process nucleosynthetic isotope and critical parent-body elemental data are unavailable for this meteorite group^5^. Furthermore, unlike the APB and EPB, the mass-dependent Si isotope composition of aubrites is chondritic, and hence clearly lighter than the BSE^49^. Thus, in Run 4, hypothetical unsampled materials were adopted as DNC endmembers. In detail, these materials were characterized by the elemental abundances and mass-dependent isotope compositions of the APB and EPB, but the mass-independent isotope compositions of ordinary and enstatite chondrites, respectively, for Unsampled Materials 1 and 2 (UM1 and UM2; see Supplementary Tables S3 and S4). As such, UM1 and UM2 are equivalent to DNC enstatite and ordinary chondrite planetesimals, respectively. With these parameters, Run 4 reproduced the isotope and elemental compositions of Mars with 53 ± 23% of DNC, 47 ± 23% of NCC, and 1 ± 1% of CC material (Table 2; Fig. 4). The conclusion that volatile-poor differentiated material, possibly unsampled by known meteorites, contributed significantly to the accretion of both Mars and Earth is in accord with the results of previous investigations, which studied the building blocks of both planets based on isotopic, chemical and geophysical constraints^5,47,50^.

The Run 4 results highlight a significant difference in the CC contributions to Earth (10 ± 3%)^5^ and Mars (1 ± 1%) (Table 2; Fig. 4), in accord with the results of previous nucleosynthetic Zn isotope investigations^11,12^ and the comprehensive isotopic study of Dauphas et al.^46^. Despite of Earth’s more significant CC contributions, the abundances of most volatile elements are higher for Mars than for Earth^6^. The results thus demonstrate that, while CCs may have played a substantial role in establishing Earth’s volatile inventory, large amounts of CC material derived from a colder, volatile-rich region of the Solar System were not required to build a volatile-rich terrestrial planet, as exemplified by Mars. Instead, the more important determining factor appears to be the accretion of undifferentiated chondritic material, regardless of whether this was derived from the inner or outer Solar System. This chondritic material, of either NCC or CC provenance, contributed a total of ~ 30% and ~ 50% to the masses of Earth and Mars, respectively, but critically provided about 90% of the Zn inventories to both planets. The remaining accreting material was comprised of differentiated, volatile-poor planetesimals, which supplied just ~ 10% of the Zn.

## Methods

All methods employed in this study were previously established and are documented in a number of publications. Below, the methods employed for sample preparation and the mass-independent Zn isotope analyses are summarized. The Supplementary Information provides details on the procedures applied for the mass-dependent Zn isotope analyses of the Martian and Lunar samples, and the numerical modeling.

### Sample preparation

Analyzed for this study were six SNC meteorites and five Lunar samples, as well the Orgueil CI1 chondrite and the geological reference material BCR-2 for quality control. Digestion and preparation of the samples and the subsequent Zn isotope measurements were carried out in the MAGIC Laboratories at the Department of Earth Science & Engineering of Imperial College London, following the procedures outlined in Martins et al.^1^. All sample preparation was conducted in ISO Class 6 clean rooms, using ISO Class 4 laminar flow benches for critical steps. The water used was of ≥ 18.2 MΩ cm quality from a Millipore purification system. All acids were purified from reagent grade stock acids by sub-boiling distillation in either quartz glass (15.3 M HNO_3_, 6 M HCl) or Teflon (28 M HF, 12 M HCl, 8.5 M HBr) stills.

The samples were completely crushed with an agate mortar and pestle and digested in Savillex Teflon beakers. The digestion procedure started with refluxing in a 2 + 1 mixture of 28 M HF + 15.3 M HNO_3_ at 120 °C for at least two days on a hotplate, followed by evaporation to dryness. This process was then repeated with 6 M HCl. Following digestion, the samples were purified using the three-stage anion exchange procedure described in Martins et al.^1^.

### Mass-independent Zn isotope measurements

The isotope analyses were conducted with a Nu Instruments Nu Plasma II multiple collector inductively coupled plasma mass spectrometer (MC-ICP-MS) at the MAGIC Laboratories. A Nu Instruments DSN 100 desolvation system fitted with glass cross flow nebulizers with solution flow rates of about 120 µl min^− 1^ were employed for sample introduction in conjunction with a CETAC ASX 112FR autosampler. The analytical procedures followed the methods described in Martins et al.^5^.

All Martian samples, as well as Orgueil and BCR-2, were analyzed using two cup configuration^1^. In contrast, the Lunar samples with more limited Zn were analyzed with only a single cup configuration and without performing a correction for Ge interferences on ^70^Zn^5^. All ion beams were monitored using Faraday cups fitted with 10^11^ Ω resistors. Each run was started by a peak centering routine, whilst each block commenced with a 60 s measurement of the electronic baselines of the Faraday collectors whilst the ion beam was deflected in the electrostatic analyzer. Data acquisition for the Martian samples encompassed 3 blocks with 20 measurement cycles of 8 s each. Due to the low Zn contents of the Lunar samples, their analyses encompassed only 2 blocks with 20 data acquisition cycles.

The samples were introduced as solutions in 0.1 M HNO_3_, typically containing 150–500 ng g^- 1^ of Zn, with instrumental sensitivity ranging between 150 and 350 V (µg ml^- 1^)^- 1^. All sample analyses were carried out with the sample–standard bracketing technique, in which sample runs were symmetrically bracketed by runs of the London Zn isotope reference material. The Zn concentrations of the latter were matched to the sample concentrations to within 10 to 15%.

The Zn isotope results are reported using the ε notation, which denotes deviations of the measured isotope ratio for a sample (sam) from the value determined for the London Zn reference standard (std) in parts per 10^4^:

$$\:{\epsilon\:}^{\text{i}/\text{j}}{Zn}_{\:}=\:\left(\frac{{\left({}^{\text{i}}Zn/{}^{\text{j}}Zn\right)}_{\text{s}\text{a}\text{m}}}{{\left({}^{\text{i}}Zn/{}^{\text{j}}Zn\right)}_{\text{s}\text{t}\text{d}}}-1\right)\:x\:{10}^{4}$$

where i and j are the atomic mass numbers of the isotopes of interest. For the results shown in Table 1 and used for the Zn mass balance modeling, j is equivalent to ^68^Zn and the ^64^Zn/^68^Zn ratio was used for internal normalization with the exponential law. Additional results were obtained with j equivalent to ^67^Zn and using ^66^Zn/^67^Zn for internal normalization. These data are reported in Supplementary Table S2, and they are employed in the multi-element modeling summarized in Fig. 4; Table 2. The 2sd and 2se data uncertainties shown in Table 1 and S2 are based on the results of n individual sample runs. For samples with *n* = 2, only a 2se error is reported, which reflects the average within-run uncertainty of the two sample runs.

## Supplementary Information

Below is the link to the electronic supplementary material.

Supplementary Material 1

Supplementary Material 2

## Data Availability

All data generated during this study are included in this published article and the supplementary information files.
