# Supplementary material for: Provenance and distribution of zinc in terrestrial planets
Source: Sci Rep. 2025 Nov 18;15:40546. doi: 10.1038/s41598-025-24419-4 (PMC12627581; doi:10.1038/s41598-025-24419-4)
Supplement: Supplementary file 2 — Supplementary Material 2 [file 41598_2025_24419_MOESM2_ESM.pdf]

Supplementary Information for

Provenance and distribution of zinc in terrestrial planets

Rayssa Martins[1,2*], Elin M. Morton[1], Yihang Huang[1], Helen M. Williams[2], Mark Rehkämper[1]

[1] Department of Earth Sciences and Engineering, Imperial College London, London, UK.

[2] Department of Earth Sciences, The University of Cambridge, Cambridge, UK.

Supplementary Methods

Mass-dependent Zn isotope measurements

The measurements of mass-dependent Zn isotope composition were carried out at the MAGIC Laboratories at the Department of Earth Science & Engineering, Imperial College London. For the Martian meteorites, the analyses employed well-established methods routinely used in our laboratory. This procedure could not be employed for the Lunar rocks, as they were completely consumed in the analyses of the mass-independent Zn isotope compositions. For these samples, more imprecise mass-dependent Zn isotope data were therefore obtained as a by-product of the mass-independent isotope analyses. Details for both procedures are supplied below.

Mass-dependent Zn isotope measurements of Martian meteorites

The mass-dependent Zn isotope data for the Martian meteorites reported in Table 1 were obtained with a double spike protocol using a Nu Plasma HR MC-ICP-MS, as described in previous studies (Bridgestock et al., 2014; Moore et al., 2017; Pickard et al., 2022). In brief, aliquots of the initial rock solutions were first equilibrated with a ^{64}Zn-^{67}Zn double spike, followed by separation of Zn from matrix elements by ion exchange chromatography with AG MP-1 anion exchange resin. The purified Zn fractions were then introduced to the MC-ICP-MS using a Cetac autosampler and a Cetac Aridus II desolvation system fitted with glass concentric nebulisers with a nominal uptake rate of 100 µL/min. The typical measurement sensitivity achieved for Zn was 100 V (µg ml^{-1})$^{-1}$. Each run involved monitoring the ^{64}Zn, ^{66}Zn, ^{67}Zn, and ^{68}Zn ion beams using Faraday cups fitted with 10^{11} Ω resistors for 60 data acquisition cycles of 5 s each. The ion beam intensities at mass numbers 62 ($^{62}Ni^{+}$) and 68.5 ($^{137}Ba^{2+}$) were also measured for correction of spectral interferences (Moore et al., 2017).

The mass dependent Zn isotope compositions of samples (sam) are reported relative to an isotope standard (std) using the $\delta^{66}Zn$ notation, defined as:

$$\delta^{66}Zn = \left(\frac{(^{66}Zn/^{64}Zn)_{\mathrm{sam}}}{(^{66}Zn/^{64}Zn)_{\mathrm{std}}} - 1\right) x\ 10^3 \qquad \text{(Eq. S1)}$$

The sample measurements were initially performed, and the $\delta^{66}Zn$ values initially calculated, relative to bracketing analyses of the double-spiked AA-ETH Zn isotope reference material, using solutions with matching Zn concentrations and ratios of spike-derived to natural Zn. To follow the convention of reporting $\delta^{66}Zn$ values relative to the JMC Lyon Zn isotope reference material, the reported values were corrected for a $\delta^{66}Zn$ offset of –0.28 between JMC Lyon Zn relative to AA-ETH Zn (Archer et al., 2017).

Mass-dependent Zn isotope measurements of Lunar samples

As the double spike approach could not be applied for the Lunar rocks due to the limited sample availability, less precise but still relevant mass-dependent Zn isotope data were obtained as a by-product of the mass-independent Zn isotope analyses. As such, the measurements employed the same analytical setup that was used for the mass-independent Zn isotope measurements. The mass-dependent Zn isotope results were obtained from the 'raw' $^{66}Zn/^{64}Zn$ isotope data measured for samples and bracketing runs of the London Zn standard solution. These 'raw' data were not corrected for mass bias by internal normalization or spectral interferences from $^{64}Ni^{+}$ or $^{132}Ba^{2+}$ ions. This approach is nonetheless feasible, as all sample analyses were directly bracketed by runs of a London Zn solution, and whereby the Zn concentrations of samples and standards were matched to better than 15%.

The $\delta^{66}Zn$ values of samples were calculated using Equation S1 relative to the average $^{66}Zn/^{64}Zn$ ratio measured for London Zn in runs directly before and after each sample run. Whilst the $\delta^{66}Zn$ data of the Lunar samples were initially determined relative to London Zn, they are reported relative to the JMC Lyon Zn reference material in Supplementary Table S1 for convenience. To this end, the values were corrected for a $\delta^{66}Zn$ offset of –0.12 for JMC Lyon Zn relative to London Zn (Moore et al., 2017). Importantly, it is recommended that the $\delta^{66}Zn$ values determined here for the Lunar samples are *not* employed for geochemical interpretations. This reflects that the measured $\delta^{66}Zn$ data are possibly slightly biased and more imprecise than dedicated mass-dependent Zn isotope compositions, due to uncorrected instabilities in the instrumental mass bias. In addition, they could also be impacted by minor mass fractionation effects incurred during samples processing (see main text for discussion).

Table S1. Mass-dependent Zn isotope data for the Lunar samples from this study and the literature (Lit), with $\varepsilon^{66}Zn$ results shown for comparison.

Sample	$\delta^{66}Zn$	2sd	$\delta^{66}Zn$ Lit	$\varepsilon^{66}Zn$	2se
70017-587	1.6	0.6	1.20	-0.11	0.07
10017-423	-6.0	0.2	-5.42	0.29	0.39
15016-254	-0.6	0.1	-1.47	-0.10	0.30
NWA 11182	0.9	0.2	-	0.18	0.19
NWA 11898	9.2	0.2	-	-0.29	0.06

The 2sd data for the $\delta^{66}Zn$ values of this study are based on the result of the individual samples runs. The literature $\delta^{66}Zn$ data are from Herzog et al. (2009) and Kato et al. (2015).

Modelling approaches

The modelling methods are similar (Zn mass balance) or identical (multi-element mass balance) to those previously employed by Martins et al. (2024).

Zn mass balance models based on ε^{66}Zn for Mars and Earth

A 10,000-trial Monte Carlo simulation of a mixture between all meteorite groups for which mass-independent Zn isotope data were available was performed employing the following equations:

$$\varepsilon^{64}Zn_{BE} = \sum_{i=1}^{n} p_i \times \varepsilon^{64}Zn_i \times f_i \quad \text{(Eq. S2)}$$

$$f_i = {r_i}/{\sum_{i=1}^{n}(p_i \times r_i)} \quad \text{(Eq. S3)}$$

where i corresponds to one of the meteorite groups. A value of either 0 or 1 was randomly assigned to p_i to determine whether each group would be included in a given trial. The mass fraction of each group, f_i, was attributed by randomly generating a number r_i between 1 and 99, and then normalizing it to the sum of the r_i values for all groups included in a trial (Eq. S3). The isotope compositions of each group were generated in a normal distribution, varying from the group mean within the respective uncertainty.

In contrast to the previous Zn mass balance models of Martins et al. (2023, 2024), the current calculations were performed with ε^{66}Zn values, determined from ^{66}Zn/^{68}Zn ratios internally normalized to ^{64}Zn/^{68}Zn. This allowed the Zn isotope data of Martins et al. (2023, 2024) and additional results from the literature (Paquet et al., 2023; Kleine et al., 2023) to be used as combined constraints for the modeling. This data compilation, available as a Supplementary Data File, provides comprehensive ε^{66}Zn results for 17 individual meteorite groups, as well as bulk silicate Mars (BSM) and the bulk silicate Earth (BSE).

Only trials which yielded results that were in accord with the mean BSM and BSE values were considered valid. For such trials, the sums of the fractions of all CC and NC groups which contributed to the solution were taken to represent the Zn mass fractions derived from each reservoir that provided the Zn inventory of Mars and Earth. Given that Zn was, at most, only slightly siderophile during the accretion of Mars and Earth, it is reasonable to assume that the ε^{66}Zn values of BSM and BSE are essentially identical to the bulk compositions of the two planets.

Multi-element mass balance models for Mars

The multi-element Monte Carlo simulations employed the following equations:

$$R_{BE} = \frac{\sum_{i=1}^{n} p_i \times R_i \times [E]_i \times f_i}{\sum_{i=1}^{n} p_i \times [E]_i \times f_i} \quad \text{(Eq. S4)}$$

$$[E]_{BE} = \sum_{i=1}^{n} p_i \times [E]_i \times f_i \quad \text{(Eq. S5)}$$

where i corresponds to the different meteorite groups included in the model. A value of either 0 or 1 was randomly assigned to p_i to define whether each group would be included in a given trial. The mass fraction of each group, f_i, was defined by randomly generating a number r_i between 1 and 20 for CCs and 1 and 99 for NCs, and then normalizing it to the sum of the r_i values for all groups included in each trial, according to Eq. S3. The CC mass fractions were purposefully assigned smaller values compared to NCs to optimize the number of valid solutions, as most previous studies suggest that the CC mass fraction is relatively limited (Lodders et al., 2000; Dauphas, 2017; Burkhardt et al., 2021; Martins et al., 2023). Notably, r values for NCs were also allowed within the CC range (1-20), such that large proportions (> 50%) of CC material were still possible, but less likely. The isotope ratios R_i were generated in a normal distribution, varying within uncertainties from their respective group means. The concentrations of the elements $[E]_i$ were randomly generated numbers between the lowest and highest measured results in the case of chondrites or estimated values for the different bulk achondrite parent bodies. Trials which yielded results that were in accord with the isotope composition (Run 1), elemental composition (Run 2) or both (Runs 3, 4) of BSM, as defined in Supplementary Tables S3 and S4, were considered valid.

The 13 meteorite groups employed in the mixing calculations, as well as the corresponding isotopic and elemental compositions, are identical to those previously employed in the multi-element mass balance model for Earth; their compositions, including uncertainties, are outlined in Supplementary Tables S3 and S4. In contrast to the Zn mass balance model, the calculations hence employ $\varepsilon^{64}Zn$ values, determined from $^{64}Zn/^{67}Zn$ ratios internally normalized to $^{66}Zn/^{67}Zn$. The $\varepsilon^{64}Zn$ data are advantageous as they are particularly precise. In addition, the approach renders the new modeling results obtained for Mars directly comparable to the data from essentially identical multi-element accretion models recently calculated for Earth (Martins et al., 2024). This conclusion stands, even though the Earth models of Martins et al. (2024) included $\varepsilon^{30}Si$ data, which are no longer considered here because the nucleosynthetic origin of these anomalies was recently called into question (Dauphas et al., 2024). This difference has no bearing on the modeling results, however, as the $\varepsilon^{30}Si$ data were not used as a condition by Martins et al. (2024) for the selection of valid trials.

During core formation, siderophile elements, including Fe, Ni, Mo and Ru, variably partition into Earth's core, depending on their metal-silicate distribution coefficients. The nucleosynthetic isotope compositions of these elements in Mars and Earth are therefore biased towards materials that were added relatively late during accretion. As Mars and Earth likely formed via heterogenous accretion (e.g., Dauphas et al., 2024), the nucleosynthetic isotope composition of siderophile elements in BSM and the BSE provide unsuitable constraints for one-stage accretion models, such as that used here. The modeling employed in this study and Martins et al. (2024) hence focused solely on lithophile element isotope data, which trace most or all of Mars' and Earth's accretion. Neodymium was not included, however, as the BSE's Nd isotope composition was possibly altered by collisional erosion and preferential loss of more incompatible elements (Frossard et al., 2022). Likewise, the models do not take ^{40}K anomalies into account, as the available results are presently limited to chondritic meteorites

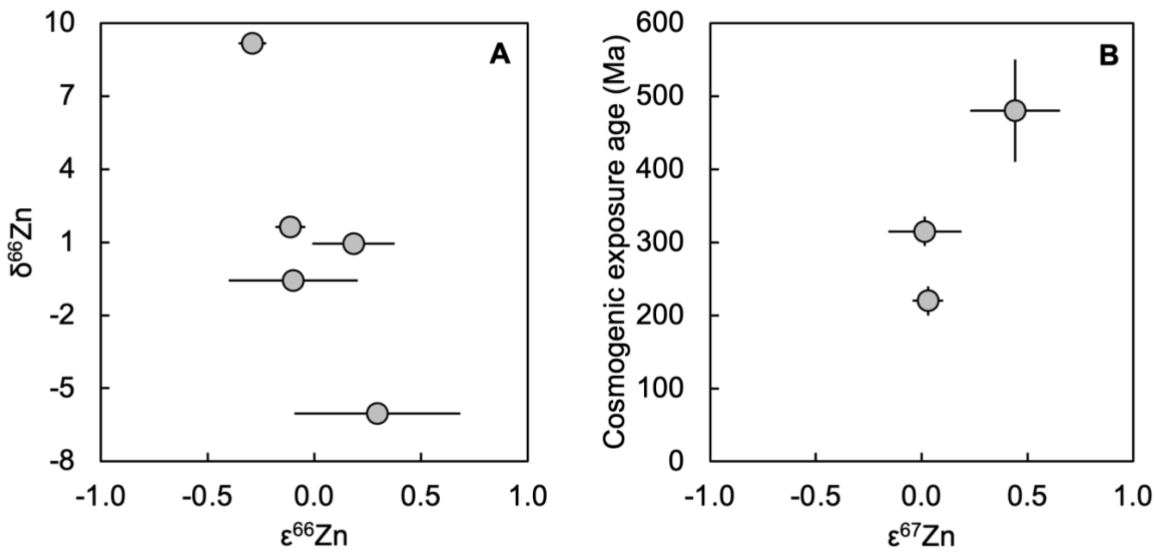

Figure S1. Mass-dependent Zn isotope compositions and cosmogenic exposure ages of the Lunar samples. Panel (A) plots the mass-dependent Zn isotope compositions, reported as δ^{66}Zn values, against the mass-independent isotope composition (ε^{66}Zn) obtained for the same samples. Panel (B) shows the cosmogenic exposure ages relative to ε^{67}Z.n

Table S2. Mass-independent Zn isotope data for Martian and Lunar samples, and Orgueil CI1 and BCR-2; the data were obtained from $^{i}Zn/^{67}Zn$ ratios employing $^{66}Zn/^{67}Zn$ for internal normalization.

**Sample name**	**Type**	**$\varepsilon^{64}Zn$**	**2sd**	**2se**	**n**	**$\varepsilon^{68}Zn$**	**2sd**	**2se**	**n**	**$\varepsilon^{70}Zn$**	**2sd**	**2se**	**n**
MIL 03346	Nakhlite	0.81	0.61	0.23	7	-0.32	0.47	0.13	14	-1.01	0.76	0.29	7
ALH 77005	Shergottite	0.44	0.30	0.11	8	-0.28	0.57	0.15	14	-0.89	0.30	0.12	6
EET 79001	Shergottite	0.57	0.40	0.16	6	-0.27	0.55	0.18	9	-1.30	0.44	0.25	3
LAR 12095	Shergottite	0.47	0.52	0.20	7	-0.33	0.30	0.09	11	-0.85	0.38	0.19	4
RBT 04262	Shergottite	0.51	0.43	0.14	10	-0.28	0.45	0.12	15	-1.15	0.55	0.28	4
ALH 84001	SNC OPX	0.56	0.60	0.17	12	-0.29	0.56	0.16	12	-0.44	0.99	0.29	12
Bulk silicate Mars		0.56	0.24	0.10	6	-0.30	0.04	0.02	6	-0.94	0.55	0.22	6
70017-587	High-Ti mare basalt	0.43	0.51	0.17	9	-0.15	0.31	0.10	9	…	…	…	…
10017-423	High-Ti ilmenite mare basalt	-0.09	1.85	0.84	5	-0.59	0.33	0.15	5	…	…	…	…
15016-254	Low-Ti olivine basalt	0.44	…	0.84	2	-0.32	…	0.40	2	…	…	…	…
NWA 11182	Feldspathic breccia	0.19	…	0.54	2	-0.63	…	0.26	2	…	…	…	…
NWA 11898	Feldspathic breccia	0.30	0.53	0.26	4	0.08	0.52	0.26	4	…	…	…	…
Lunar mean		0.25	0.40	0.18	5	-0.16	0.56	0.25	5	…	…	…	…
Orgueil	CI1	-1.09	0.60	0.14	18	0.45	0.29	0.07	18	1.59	1.02	0.24	18
BCR-2	Terrestrial	0.00	0.64	0.08	70	-0.05	0.43	0.04	107	-0.02	0.92	0.11	73

n denotes the total number of individual analytical runs for a given sample, for one or several powder/digest solution aliquots. The reported 2sd and 2se uncertainties for samples with n > 2 are based on the results obtained for n individual runs; for samples with n = 2, only a 2se error is reported, which reflects the average within-run uncertainty of the two sample runs.

Table S3. Mean nucleosynthetic isotope compositions that were used in the multi-element mass balance modelling.

**Group**		$\Delta^{17}O$	±	$\varepsilon^{48}Ca$	±	$\varepsilon^{50}Ti$	±	$\varepsilon^{54}Cr$	±	$\varepsilon^{64}Zn$	±	$\varepsilon^{84}Sr$	±	$\varepsilon^{96}Zr$	±	$\delta^{30}Si$	±	$\delta^{25}Mg$	±
CI	CC	0.46	0.12	2.09	0.03	1.90	0.08	1.47	0.09	-0.93	0.20	0.33	0.09	0.52	0.35	-0.44	0.17	-0.100	0.010
CM	CC	-3.09	0.54	2.62	1.04	2.99	0.18	1.00	0.11	-1.15	0.20	0.40	0.21	0.90	0.29	-0.50	0.05	-0.145	0.010
CO	CC	-4.38	0.23	3.87	0.56	3.49	0.35	0.83	0.16	-0.57	0.22	0.47	0.16	0.80	0.25	-0.46	0.07	-0.158	0.010
CV	CC	-3.63	0.40	3.24	1.36	3.28	0.14	0.88	0.08	-0.83	0.08	0.79	0.07	1.01	0.17	-0.47	0.07	-0.134	0.035
H	NC	0.93	0.04	-0.04	0.36	-0.57	0.09	-0.37	0.03	0.47	0.06	0.03	0.40	0.48	0.19	-0.45	0.02	-0.145	0.025
L	NC	1.05	0.03	-0.26	0.08	-0.64	0.02	-0.34	0.10	0.81	0.34	0.03	0.11	0.37	0.15	-0.45	0.04	-0.147	0.005
LL	NC	1.20	0.05	-0.32	0.09	-0.67	0.03	-0.40	0.06	0.67	0.02	-0.15	0.15	0.31	0.03	-0.43	0.04	-0.150	0.004
OC Mean		*1.06*	*0.22*	*-0.21*	*0.24*	*-0.62*	*0.08*	*-0.37*	*0.05*	*0.65*	*0.28*	*-0.03*	*0.17*	*0.36*	*0.11*	*-0.45*	*0.02*	*-0.147*	*0.005*
EH	NC	-0.04	0.10	-0.20	0.23	-0.15	0.03	0.05	0.06	0.84	0.13	-0.09	0.06	0.09	0.12	-0.72	0.06	-0.133	0.007
EL	NC	0.03	0.05	-0.25	0.13	-0.25	0.06	0.03	0.05	0.63	0.25	0.04	0.05	0.15	0.06	-0.58	0.03	-0.133	0.007
EC Mean		*-0.01*	*0.08*	*-0.22*	*0.05*	*-0.20*	*0.10*	*0.04*	*0.02*	*0.74*	*0.21*	*-0.03*	*0.12*	*0.12*	*0.06*	*-0.65*	*0.13*	*-0.13*	*0.00*
Angrites	NC	-0.08	0.05	-1.06	0.18	-1.16	0.02	-0.41	0.06	0.89	0.42	0.00	0.09	0.50	0.05	-0.33	0.06	-0.079	0.000
HEDs	NC	-0.29	0.07	-1.37	0.26	-1.24	0.03	-0.69	0.06	1.39	0.39	-0.07	0.09	0.41	0.09	-0.43	0.03	-0.111	0.013
BSE		0.002	0.004	0.01	0.01	0.01	0.01	0.09	0.12	0.02	0.11	-0.07	0.15	0.05	0.05	-0.29	0.08	-0.121	0.005
BSM		0.25	0.06	-0.20	0.03	-0.42	0.17	-0.16	0.03	0.55	0.09	-0.25	0.15	0.28	0.03	-0.46	0.07	-0.13	0.03
UM1	NC	1.06	0.22	-0.21	0.24	-0.62	0.08	-0.37	0.05	0.65	0.28	-0.03	0.17	0.36	0.11	-0.38	0.10	-0.095	0.032
UM2	NC	-0.01	0.08	-0.22	0.05	-0.20	0.10	0.04	0.02	0.74	0.21	-0.03	0.12	0.12	0.06	-0.38	0.10	-0.095	0.032

The mean values for Mars were compiled from Magna et al. (2017), Liebske & Khan (2019), Losno et al. 2022) and Rüfenacht, M. et al. (2023). All other data adapted from Martins et al. (2024).

Table S4. Compiled elemental concentration data for Mars and the meteorite classes considered in the modelling.

	**Bulk Mars**	**CI**	**CM**	**CO**	**CV**	**H**
Na (%)	0.37 – 0.87	0.25 – 0.58	0.087 – 0.46	0.365 – 0.41	0.238 – 0.316	0.591 – 0.659
Mg (%)	16.46 – 19.90	9.5 – 10.5	11.5 – 12.4	10.8 – 13	12.5 – 14.5	13.8 – 14.4
Al (%)	1.53 – 1.90	0.80 – 0.86	1.1 – 1.24	1.11 – 1.43	1.42. -1.75	1.1 – 1.2
Si (%)	20.50 – 22.20	10.5	12.9	15.9	15.6	16.9
P (μg g^{-1})	675 – 740	845 – 1108	900 – 1108	1010 – 1060	990 – 1041	1080
Ca (%)	1.43 – 2.06	0.78 – 1.01	1.01 – 1.55	1.28 – 1.58	1.59 – 1.90	1.17 – 1.33
K (μg g^{-1})	305 – 360	407 – 608	206 – 518	272 – 345	251 – 310	727 – 860
Ti (μg g^{-1})	600 – 1010	381 – 458	520 – 664	601 -656	753 – 980	600
Fe (%)	11.40 – 14.10	18.2 – 21.3	21 – 23.6	21.7 – 24.8	22.2 – 23.8	26.4 – 29.7
Mn (μg g^{-1})	2840 – 4800	1900 – 2061	1700 – 1920	1475 – 1650	1334 – 1465	2180 – 2430
O (%)	43.2	46	43.2	37	37	35.7
Cr (μg g^{-1})	4640 – 6000	2575 – 3001	3033 – 3478	3111 – 3550	3253 – 3603	3460 – 3870
Zn (μg g^{-1})	19 – 83	312 – 391	182 – 214	100 – 119	112 – 127	41 – 55
Sr (μg g-1)	13.5 – 17.6	3.87 – 5	4.48 – 10.1	4.92 – 12.7	5.85 – 15.3	10
Zr (μg g^{-1})	13.5 – 17.6	3.8-4	8	7.8	8.3	6.3

Continued on next page.

Table S4. Continued.

	L	LL	EH	EL	EPB	APB	UM1/2
Na (%)	0.58 - 0.731	0.576 - 0.728	0.474 - 0.78	0.51 - 0.63	0.104 - 0.54	0.02	0.02 - 0.54
Mg (%)	14.1 - 15.2	14.7 - 15.5	9.6 11.9	13.3 - 14.6	15 - 18.4	12.0	12.0 - 18.4
Al (%)	1.17 - 1.26	1.16 - 1.31	0.74 - 0.87	1 - 1.14	1.2 - 1.9	1.43	1.2 - 1.43
Si (%)	18.5	18.9	16.7	18.6	20.9 - 21.5	13.1	13.1 - 21.5
P (μg g^{-1})	950	850	2000	1170	1877	938	938 - 1877
Ca (%)	1.23 - 1.38	1.18 - 1.39	0.65 - 1	0.85 - 1.2	1.3 - 2.14	1.50	1.3 - 2.14
K (μg g^{-1})	505 - 950	637 - 922	402 - 1240	600 - 845	30 - 54	12	12 - 54
Ti (μg g^{-1})	630	620	450	580	347.4 - 830	599.0	347.4 - 830
Fe (%)	20.3 - 24.5	17.6 - 21.2	24.9 - 31.5	19.5 - 27	11.1 - 29.2	19.4	11.1 - 29.2
Mn (μg g^{-1})	2410 - 2700	2490 - 2720	1820 - 2650	1120 - 2560	1394 - 4336	1549	1394 - 4336
O (%)	37.7	40	28	31	34.48 - 42.65	34.5	34.48 - 42.65
Cr (μg g^{-1})	3630 - 3940	3630 - 4060	2820 - 3870	2460 - 3870	1916 - 4611	2737	1916 - 4611
Zn (μg g^{-1})	43.5 - 63	41 - 60.6	21 - 342	13 - 26	0.11 – 8.9	5 – 12	0.11 - 12
Zr (μg g^{-1})	11.1	11.1	7.2	8.2	7.9	6.32 – 7.1	6.32 – 7.9
Sr (μg g^{-1})	5.9	5.9	4.9	5.2	16.6	10	10 – 16.6

Martian data compiled from Wänke(1994), Lodders & Fegley (1997), Sanloup et al. (1999), Taylor (2013) and Yoshizaki & McDonough (2022). All other data are adapted from Martins et al. (2024).

Bibliography

Archer, C. *et al.* Inter-calibration of a proposed new primary reference standard AA-ETH Zn for zinc isotopic analysis. *J Anal At Spectrom* **32**, 415-419 (2017).

Bridgestock, L. J. *et al.* Unlocking the zinc isotope systematics of iron meteorites. *Earth Planet Sci Lett* **400**, 153–164 (2014).

Burkhardt, C. *et al.*, Terrestrial planet formation from lost inner solar system material. *Sci Adv* **7**, eabj7601 (2021).

Dauphas, N. The isotopic nature of the Earth's accreting material through time. *Nature* **541**, 521-524 (2017).

Dauphas, N., Hopp, T. & Nesvorný, D. Bayesian inference on the isotopic building blocks of Mars and Earth. *Icarus* **408**, 115805 (2024).

Frossard, P., Israel, C., Bouvier, A., Boyet, M. Earth's composition was modified by collisional erosion. *Science* **377**, 1529-1532 (2022).

Herzog, G. F., Moynier, F., Albarède, F. & Berezhnoy, A.A. Isotopic and elemental abundances of copper and zinc in lunar samples, Zagami, Pele's hairs, and a terrestrial basalt. *Geochim Cosmochim Ac* **73,** 5884-5904 (2009).

Kato, C., Moynier, F., Valdes, M.C., Dhaliwal, J.K., Day, J. M. D. Extensive volatile loss during formation and differentiation of the Moon. *Nat Commun* **6**, 7617 (2015).

Kleine, T., Steller, T., Burkhardt, C. & Nimmo, F. An inner solar system origin of volatile elements in Mars. *Icarus* **397**, 115519 (2023).

Liebske, C. & Khan, A. On the principal building blocks of Mars and Earth. *Icarus* **322**, 121-134 (2019).

Lodders, K. An oxygen isotope mixing model for the accretion and composition of rocky planets. *Space Sci Rev* **92**, 341-354 (2000).

Losno, D., Fitoussi, C. & Bourdon, B. Early differentiation processes on Mars inferred from silicon isotopes. *Geochim Cosmochim Ac* **338**, 11-23 (2022).

Magna, T., Hu, Y., Teng, F.-Z. & Mezger, K. Magnesium isotope systematics in Martian meteorites. *Earth Planet Sci Lett* **474**, 419-426 (2017).

Martins, R., Kuthning, S., Coles, B. J., Kreissig, K. & Rehkämper, M. Nucleosynthetic isotope anomalies of zinc in meteorites constrain the origin of Earth's volatiles. *Science* **379**, 369-372 (2023).

Martins, R. *et al.* Primitive asteroids as a major source of terrestrial volatiles. *Sci Adv* **10**, eado4121 (2024).

Moore, R. E. T., Larner, F., Coles, B. J. & Rehkämper, M. High precision zinc stable isotope measurement of certified biological reference materials using the double spike technique and multiple collector-ICP-MS. *Anal Bioanal Chem* **409**, 2941–2295 (2017).

Paquet, M., Sossi, P. A. & Moynier, F. Origin and abundances of volatiles on Mars from the zinc isotopic composition of Martian meteorites. *Earth Planet Sci Lett* **611**, 118126 (2023).

Pickard, H. *et al.* The cadmium and zinc isotope compositions of the silicate Earth – Implications for terrestrial volatile accretion. *Geochim Cosmochim Ac* **338**, 165-180 (2022).

Rüfenacht, M. *et al.* Genetic relationships of solar system bodies based on their nucleosynthetic Ti isotope compositions and sub-structures of the solar protoplanetary disk. *Geochim Cosmochim Ac* **355**, 110-125 (2023).
